# The live birth rate of vitrified oocyte accumulation for managing diminished ovarian reserve: a retrospective cohort study

**DOI:** 10.1186/s13048-023-01128-y

**Published:** 2023-03-03

**Authors:** Kuan-Sheng Lee, Ming-Huei Lin, Yuh-Ming Hwu, Jia-Hwa Yang, Robert Kuo-Kuang Lee

**Affiliations:** 1 Present address, Department of Obstetrics & Gynecology, Division of Reproductive Endocrinology and Infertility, MacKay Memorial Hospital, No 92, Sec 2, Zhong-San North Road, Taipei, 104 Taiwan; 2 MacKay Junior College of Medicine, Nursing and Management, New Taipei City, 251 Taiwan; 3 Department of Medicine, MacKay Medical College, New Taipei City, 252 Taiwan; 4 Department of Medical Research, Mackay Memorial Hospital, Taipei, 104 Taiwan; 5 Taipei Fertility Center (TFC), Taipei, Taiwan; 6 Taiwan Public Health Association, Taipei, 100 Taiwan

**Keywords:** Diminished ovarian reserve (DOR), Fresh oocyte, Vitrified oocyte accumulation, Live birth rate (LBR)

## Abstract

**Background:**

Vitrified M-II oocyte accumulation for later simultaneous insemination has been used for managing POR. Our study aimed to determine whether vitrified oocyte accumulation strategy improves live birth rate (LBR) for managing diminished ovarian reserve (DOR).

**Methods:**

A retrospective study included 440 women with DOR fulfilling Poseidon classification groups 3 and 4, defined as the presence of serum anti-Müllerian hormone (AMH) hormone level < 1.2 ng/ml or antral follicle count (AFC) < 5, from January 1, 2014, to December 31, 2019, in a single department. Patients underwent accumulation of vitrified oocytes (DOR-Accu) and embryo transfer (ET) or controlled ovarian stimulation (COS) using fresh oocytes (DOR-fresh) and ET. Primary outcomes were LBR per ET and cumulative LBR (CLBR) per intention to treat (ITT). Secondary outcomes were clinical pregnancy rate (CPR) and miscarriage rate (MR).

**Results:**

Two hundred eleven patients underwent simultaneous insemination of vitrified oocyte accumulation and ET in the DOR-Accu group (maternal age: 39.29 ± 4.23 y, AMH: 0.54 ± 0.35 ng/ml), and 229 patients underwent COS and ET in the DOR-fresh group (maternal age: 38.07 ± 3.77 y, AMH: 0.72 ± 0.32 ng/ml). CPR in the DOR-Accu group was similar in the DOR-fresh group (27.5% vs. 31.0%, *p* = 0.418). However, MR was statistically higher (41.4% vs. 14.1%, *p* = 0.001), while LBR per ET was statistically lower (15.2% vs. 26.2%, *p* < 0.001) in the DOR-Accu group. There is no difference in CLBR per ITT between groups (20.4% vs. 27.5%, *p* = 0.081). The secondary analysis categorized clinical outcomes into four groups regarding patients’ age. CPR, LBR per ET, and CLBR did not improve in the DOR-Accu group. In the group of 31 patients, accumulated vitrified metaphase II (M-II) oocytes reached a total number of ≥ 15, and CPR improved among the DOR-Accu group (48.4% vs. 31.0%, *p* = 0.054); however, higher MR (40.0% vs. 14.1%, *p* = 0.03) resulted in similar LBR per ET (29.0% vs. 26.2%, *p* = 0.738).

**Conclusions:**

Vitrified oocyte accumulation for managing DOR did not improve LBR. Higher MR resulted in lower LBR in the DOR-Accu group. Therefore, the vitrified oocyte accumulation strategy for managing DOR is not clinically practical.

**Trial registration:**

The study protocol was retrospectively registered and was approved by Institutional Review Board of Mackay Memorial Hospital (21MMHIS219e) on August 26, 2021.

## Introduction

Poor ovarian responders (POR) or DOR are encountered during infertility treatment, and poor prognosis during in vitro fertilization (IVF) treatment is ascribed to it [1–3]. More oocytes were needed to optimize the chance of pregnancy for POR or DOR women. Therefore, the conception of creating a large stock of oocytes by accumulating vitrified M-II oocytes with multiple ovarian stimulation cycles was proposed. It theoretically helps to increase the chances of live birth by making POR or DOR patients’ number of MII oocytes a “normal responder-like” status. Thus, a vitrified M-II oocyte accumulation strategy for later simultaneous insemination has been used for managing POR since two studies reported improved IVF outcomes [4, 5]. Current data from vitrified oocyte accumulation to manage POR women were pooled together with fresh oocytes. However, it caused difficulty in determining the contribution of CPR and LBR from pooling together vitrified oocytes or fresh oocytes. Therefore, we decided whether the vitrified oocyte accumulation strategy from DOR women improves LBR in assisted reproduction technology (ART).

Numerous randomized controlled trials and prospective and retrospective studies have shown that cryopreserved oocytes provide reproductive outcomes comparable to fresh oocyte use [6–21]. However, extensive cohort analysis has indicated lower reproductive results using vitrified donor oocytes rather than fresh donor oocytes [22–24]. In addition, alterations in gene expression and reduced mitochondrial DNA content in vitrified and thawed oocytes have been found [25–27]. Despite extensive literature examining cryopreserved oocyte quality clinical characteristics, there still needs to be more data regarding whether ART outcomes of using vitrified and thawed oocytes from DOR women are comparable to those using fresh oocytes from DOR women.

Therefore, the study objective was to evaluate whether the vitrified oocyte accumulation for later simultaneous insemination improves LBR to manage DOR.

## Materials and methods

### Study design

A medical record review was performed for the DOR women who underwent COS and ET using vitrified oocyte accumulation for later simultaneous insemination or fresh oocytes at the Infertility Division of Mackay Memorial Hospital in Taipei City, Taiwan from January 1, 2014, to December 31, 2019. The patients were followed during treatment at our center for at least one year until either treatment discontinuation or one live infant delivery. The study protocol was approved by the Institutional Review Board of Mackay Memorial Hospital (21MMHIS219e).

### Study participants

DOR was made in accordance with the Poseidon (Patient-Oriented Strategies Encompassing IndividualizeD Oocyte Number) classification groups 3 and 4 [28], defined as the presence of low serum AMH hormone level (< 1.2 ng/ml) or low AFC (< 5) at time of ovarian stimulation initiation. All patients who met the criteria of Poseidon Groups 3 and 4 and had at least one embryo created intended to transfer during the current cycle were included.

Exclusion criteria were coexisting endocrine disorders (diabetes mellitus, untreated hyperprolactinemia, untreated thyroid dysfunction, congenital adrenal hyperplasia, and Cushing’s syndrome), untreated hydrosalpinx, and uterine anomaly confirmed either by hysterosalpingography or hysteroscopy. After applying the exclusion criteria, 440 DOR women who underwent fresh ET were included for final analysis. The study group included 211 patients with vitrified M-II oocyte accumulation for later simultaneous insemination. This group was named “diminished ovarian reserve, accumulation of vitrified oocytes” (DOR-Accu). In this group, we used double stimulation in the same ovarian cycle to maximize the oocyte number retrieved in a short time frame [29, 30]. After oocyte retrieval, all mature oocytes were vitrified and stored. Then, luteal phase ovarian stimulation following oocyte retrieval was performed based on the number of remainder AFC. The decision about whether to stop oocyte accumulation was based on two factors as follows: (1) the vitrified M-II oocytes’ total number reaches 10–15, which was expected to maximize the LBR [31–33], and (2) the patient’s own decision.

The control group included 229 DOR patients who underwent GnRH antagonist protocol, whose fresh mature oocytes were inseminated, and subsequent ET was named “diminished ovarian reserve, fresh oocytes” (DOR-fresh). Surplus embryos in both groups had been vitrified and transferred in their following cycle until surplus embryos were exhausted or the patient got at least one live infant delivery.

### Ovarian stimulation protocols

People in the DOR-fresh group using GnRH antagonist protocol. Patients using GnRH antagonist protocol started 300–450 IU recombinant FSH (Gonal-F; Merck Serono) or follitropin β (Puregon®; Organon) either alone or in combination with human menopausal gonadotropin (Menopur; Ferring) on days 2–3 of the menstrual cycle. Subcutaneous cetrorelix (Cetrotide; Merck Serono) 0.25 mg was introduced daily as soon as follicles reached 14 mm in diameter until trigger day. The gonadotropin dosage was adjusted every 2–3 days in accordance with follicle growth. When the leading follicle reached 16–18 mm in diameter, final oocyte maturation was induced with the combination of 250 μg recombinant hCG (Ovidrel; Merck Serono) and 0.2 mg triptorelin (Decapeptyl; Ferring). Transvaginal oocyte retrieval was performed under transvaginal ultrasound guidance 35 to 36 h after triggering.

People in the DOR-Accu group used double stimulations in the same menstrual cycle with gonadotropins with or without clomiphene or letrozole combination. In follicle phase stimulation, clomiphene citrate 150 mg/day or letrozole 7.5 mg/day were given on days 2–3 of the menstrual cycle. Gonadotropin 150–450 IU/day was added later when three or more follicles reached 10 mm in diameter, and 0.25 mg subcutaneous cetrorelix (Cetrotide; Merck Serono) was administered in the presence of 14 mm follicle until trigger day. When the leading follicle reached 16–18 mm in diameter, oocyte maturation was triggered, and oocytes were retrieved as the DOR-fresh group. Transvaginal ultrasound was performed after oocyte retrieval, and clomiphene citrate 150 mg/day or letrozole (7.5 mg/day) was given in the presence of at least one AFC. Gonadotropin 150–450 IU/day was added when three or more follicles reached 10 mm until trigger day. Administration of GnRH antagonist, the trigger of oocyte maturation, and oocyte retrievals were carried out as the follicular phase.

The oocytes from the DOR-Accu group were vitrificated by a 3-step gradient cryoprotectant loading process using Cryotec Vitrification Method® (REPROLIFE Inc. 2–5-3-9F Shinjuku, Tokyo, Japan). The oocytes were equilibrated for 12–15 min in a 0.3 ml equilibration solution. Then oocytes were washed in a 0.3 ml vitrification solution (VS) for 30–40 secs and replaced with new 0.3 ml VS for another 10–20 secs. In the next step, oocytes were loaded on the Cryotec seat with minimum (0.01–0.1 μl) VS volume and immediately submerged Cryotec into liquid nitrogen directly and then covered with a cap. All procedure was performed at room temperature (25 °C–27 °C) with media being prepared at least one hour in advance.

Firstly, the warming procedure (REPROLIFE Inc. 2–5-3-9F Shinjuku, Tokyo, Japan) started with Cryotec removal from liquid nitrogen and immersion in 1 ml warming solution (TS) for 1 min. Secondly, oocytes were transferred into a 0.3 ml dilution solution for 3 min. Thirdly, oocytes were equilibrated in 0.3 ml washing solution (WS) for another 5 min and replaced with a new 0.3 ml WS for another 1 min. Finally, oocytes were incubated in culture media for two hours at 37 °C, 6.0% CO2, and 5% O2 before intracytoplasmic sperm injection (ICSI). TS was placed in an incubator at 37 °C at least three hours before use, and the other two were prepared for one hour at room temperature (25 °C–27 °C) in advance. All warming procedure was also performed at room temperature.

### Insemination and embryo transfer

Warmed oocytes were cultured for three hours before ICSI. Fresh oocytes were denudated immediately following oocyte retrieval. In vitro insemination procedures were performed 38 to 39 h post triggering for fresh oocytes, with exceptions in male factor, in which ICSI was performed instead. In addition, assisted hatching was performed to improve embryo capacity to implant.

### Endometrial preparation, luteal phase support, and pregnancy confirmation

In the DOR-fresh group, patients received 50 mg progesterone on oocyte retrieval day. Then, plus 125 μg intramuscular injection of recombinant hCG (Ovidrel; Merck Serono) every three days plus daily vaginal supplementation of 90 mg vaginal progesterone gel (Crinone 8%; Merck Serono) or oral 10 mg dydrogesterone (Duphaston®; Abbott Biologicals) every eight hours plus 2 mg oral estradiol (E2) valerate (Progynova; Synmosa Biopharma Corporation) every eight hours plus vaginal supplementation of 90 mg vaginal progesterone gel (Crinone 8%; Merck Serono) every 12 h started on day one after oocyte retrieval as following luteal phase support.

In the DOR-Accu group, endometrial preparation was started with oral estradiol (E2) valerate (Progynova; Synmosa Biopharma Corporation) 8 mg daily on days 1–3 of the menstrual cycle. After seven days of oral estrogen supplementation, we started to perform an ultrasound to measure the endometrial thickness. If the endometrium was thinner than 7 mm on day 8, we increased the estrogen dose to 12 mg daily, followed by a reevaluation with ultrasound on day 13 of estrogen supplementation. If endometrium had reached at least 7 mm, all vitrified oocytes were warmed and inseminated by ICSI. We continued oral estrogen 8 mg daily and started 90 mg vaginal progesterone gel (Crinone 8%; Merck Serono) every 12 h plus 10 mg oral dydrogesterone (Duphaston®; Abbott Biologicals) every eight hours since the inseminated oocytes day (day 0). If the endometrium had reached 7 mm or more, oocyte thawing, endometrial preparation, and luteal phase support started on day 1 after thawing oocytes were done, as mentioned before.

After oocyte retrieval or oocyte thawing, serum β-hCG was measured 14 days later, or urine hCG was checked 16 days later. Serum β-hCG above 5 mIU/mL or urine hCG above 25 mIU/mL was a positive pregnancy. Luteal support was continued until the 10th week of gestation.

### Primary and secondary outcomes

The study’s primary outcomes were LBR per ET and CLBR per ITT. Secondary outcomes included oocyte survival rate, fertilization rate, the mean number of embryos transferred, CPR per ET, implantation rate (IR), MR per pregnancy, the mean number of surplus vitrified embryos, and CLBR per OPU. A subgroup analysis was conducted on cases with ≥ 10 and ≥ 15 M-II oocytes. To control repeat ET confounding factors, we only include the last cycle with ET for final analysis if patients underwent a repeat IVF cycle or repeat vitrified oocyte accumulation for later simultaneous insemination. Fertilization was assessed 16–18 h after insemination by visualization of two pronuclei and two polar bodies. CPR was defined as the presence of at least one gestational sac between the 5th and 6th weeks of gestation in an ultrasound per ET. IR was calculated by dividing the total number of gestational sacs detected by the total number of transferred embryos. MR was defined as a spontaneous loss of all intrauterine pregnancies before the completed 20-week gestational age. LBR was defined as the number of delivery resulting in a live-born neonate who reached 20-week gestational age per ET. CLBR calculated live birth until either cryotransfers of all embryos or one live infant delivery. For the OPU number, we only counted retrievals; at least one M-II oocyte was available for later insemination.

### Statistical analysis

Statistical analysis was performed with R software, version 3.3.1 (R Project for Statistical Computing, Vienna, Austria). Differences in demographics among the two groups were assessed with Student’s *t-*test, chi-square, or Fisher’s test, and results for continuous variables were presented as the mean and standard deviation, whereas categorical variables were expressed as percentages. Odds ratios (OR) and corresponding 95% confidence intervals (CIs) were calculated by logistic regression analysis with relevant significant variables adjusted to assess the effect of age, strategy, AMH, number of embryos transferred, and ET day on clinical outcomes. The 95% CIs for differences between proportions were calculated for LBR. Statistical significance was defined at a 95% level (*P* < 0.05).

## Results

Table 1 showed mean age at ART start was older in the DOR-Accu group (39.29y vs. 38.07y, *p* < 0.001), and mean AMH was lower in the DOR-Accu group (0.54 ng/ml vs. 0.72 ng/ml, *p* < 0.001) than the DOR-fresh group. There is no difference in reason for ART between groups. In the DOR-fresh group, 229 women obtained 809 mature oocytes, resulting in a mean of 3.53 M-II oocytes for insemination. The DOR-Accu group consisted of 211 patients who received 1,130 stimulation and oocyte retrieval cycles, resulting in a mean of 5.36 cycles per woman. A total number of 2,089 M-II oocytes were retrieved and vitrified. These oocytes were warmed, and 1,791 survival M-II *oocytes* (survival rate: 85.7%) were submitted to ICSI. Fertilization rates, CPR, and IR in the DOR-Accu group were similar to the DOR-fresh group. The mean number of embryos transferred per cycle was more in the DOR-Accu group (2.96 vs. 2.14, *p* < 0.001). MR was statistically higher (41.4% vs. 14.1%, *p* < 0.001) and LBR per ET was statistically lower (15.2% vs. 26.2%,* p* < 0.004) belonging to DOR-Accu group. No statistical differences were found between the groups regarding CLBR per ITT (20.4% vs. 27.5%, *p* = 0.081) despite more mean surplus vitrified embryos per patient (1.18 embryos vs. 0.24 embryos, *p* < 0.001) for additional cryotransfers in the DOR-Accu group. CLBR per OPU is statistically higher in the DOR-fresh group (3.8% vs. 27.5%, *p* < 0.001).

**Table 1:** Patient and cycle characteristics of the strategy for managing DOR compared between fresh M-II oocytes and accumulation of vitrified M-II oocytes

Variable	DOR-Accu	DOR-fresh	*p*
Number of patients	211	229	
Number of OPU	1130	229	
OPU /patient Mean (SD)	5.36(2.71)	1.00(0.00)	< 0.001*
Maternal age at ART start Mean (SD)	39.29(4.23)	38.07(3.77)	0.001*
Maternal age at ET Mean (SD)	40.23(4.30)	38.07(3.77)	< 0.001*
AMH Mean (SD)	0.54(0.35)	0.72(0.32)	< 0.001*
Reason for ART (%)			
DOR	211(100)	229(100)	1.00
Male factor	81(38.4)	98(42.8)	0.347
Tubal factor	39(18.5)	48(21.0)	0.515
Endometriosis	52(24.6)	52(22.7)	0.633
Unexplained or others	90(42.7)	93(40.6)	0.664
Number of total warmed or fresh M-II	2089	809	
M-II oocytes /patient Mean (SD)	9.90 (4.77)	3.53 (1.57)	< 0.001*
Number of survival warmed or fresh M-II	1791	809	
Number of the fertilized egg	1317	582	
Fertilization of survival and fresh egg % (SD)	75.18(20.98)	75.29(24.69)	0.958
ET Day (%)			< 0.001*
Day 2–3	174(82.5)	220(96.1)	
Day 4–5	37(17.5)	9(3.9)	
Number of fresh ET	211	229	
Number of embryos transferred Mean (SD)	2.96(0.95)	2.14(0.87)	< 0.001*
Pregnancy /ET (%)	58(27.5)	71(31.0)	0.418
Implantation rate % (SD)	12.99(25.16)	17.47(29.52)	0.089
Miscarriage /pregnancy (%)	24(41.4)	10(14.1)	< 0.001*
Ectopic /pregnancy (%)	1(1.7)	0(1.4)	1.00
Stillbirth /ET (%)	1(1.7)	1(1.4)	1.00
Live birth /ET (%)	32(15.2)	60(26.2)	0.004*
Number of surplus vitrified embryo embryo	248	55	
Surplus vitrified embryo /patient Mean (SD)	1.18(1.80)	0.24(0.65)	< 0.001*
Cumulative live birth /ITT (%)	43(20.4)	63(27.5)	0.081
Cumulative live birth /OPU (%)	43(3.8)	63(27.5)	< 0.001*

Data are mean ± standard deviation or n (%) and compared among groups using Student’s *t-*test, chi-square test, or Fisher’s test for *P*-value. Abbreviations: DOR-Accu = diminished ovarian reserve, accumulation of vitrified oocytes; *DOR-fresh* diminished ovarian reserve, fresh oocytes, *DOR* diminished ovarian reserve, *OPU* ovum pick-up, *ART* assisted reproduction technology, *ET* embryo transfer, *AMH* Anti-Müllerian hormone, *M-II* metaphase II, *ITT* intention to treat

* *p* < 0.05

Table 2 showed clinical outcomes were categorized into four groups regarding patients’ age. Available M-II *oocytes*, embryos transferred per ET, and the mean number of surplus vitrified embryos per patient were more in the DOR-Accu group than in the DOR-fresh group in all age groups. However, there is no difference in CPR between groups. Higher MR in the DOR-Accu group aged 35–37 (33.3% vs. 7.7%, *p* = 0.048) and 38–40 (56.2% vs. 17.4%, *p* = 0.011) results in lower LBR per ET. MR of women over 40 in both groups was similarly high, leading to low LBR. Similar fertilization, IR, and CLBR from DOR-Accu and DOR-fresh groups were observed in all age groups. CLBR per OPU was statistically higher in the DOR-fresh group and was similarly poor in both groups aged over 40.

**Table 2:** Clinical outcomes of the strategy for managing DOR according to patient’s age compared between fresh M-II oocytes and accumulation of vitrified M-II oocytes

	< 35			35–37			38–40			> 40		
	Accu	Fresh	*p*	Accu	Fresh	*p*	Accu	Fresh	*p*	Accu	Fresh	*p*
Number of patients	28	36		38	55		57	74		88	64	
Number of OPU	117	36		192	55		301	74		519	64	
OPU /patient Mean (SD)	4.18 (2.13)	1	< 0.001*	5.05 (2.68)	1	< 0.001*	5.28 (2.32)	1	< 0.001*	5.91 (2.97)	1	< 0.001*
Age at ART start (y), mean (SD)	31.89 (2.11)	31.86 (2.21)	0.954	36.03 (0.79)	36.09 (0.78)	0.696	39.11 (0.75)	38.84 (0.81)	0.055	43.17 (1.90)	42.36 (1.60)	0.01*
M-II oocytes /patient Mean (SD)	8.50 (3.92)	3.64 (1.44)	< 0.001*	10.26 (5.33)	3.91 (1.34)	< 0.001*	10.00 (4.32)	3.64 (1.67)	< 0.001*	10.12 (5.03)	3.03 (1.62)	< 0.001*
Fertilization of fresh survival or M-II % (SD)	77.08 (22.51)	74.26 (24.00)	0.633	75.07 (16.25)	77.03 (22.16)	0.643	74.51 (24.18)	74.85 (23.64)	0.936	75.05 (20.37)	74.90 (28.54)	0.968
Number of fresh ET	28	36		38	55		57	74		88	64	
Embryos transferred Mean (SD)	2.18 (0.82)	1.81 (0.58)	0.037*	2.58 (0.89)	2.33 (0.67)	0.123	3.02 (0.9)	2.27 (0.96)	< 0.001*	3.33 (0.85)	2.00 (0.99)	< 0.001
Pregnancy /ET (%)	10(35.7)	16(44.4)	0.481	18(47.4)	26(47.3)	0.993	16(28.1)	23(31.1)	0.709	14(15.9)	6(9.4)	0.239
Implantation rate % (SD)	25.00 (39.93)	30.56 (38.32)	0.574	24.78 (32.68)	26.67 (32.01)	0.782	12.28 (21.08)	15.43 (27.12)	0.471	4.55 (10.64)	4.56 (16.19)	0.996
Miscarriage /ET (%)	1(10.0)	1(6.2)	1.000	6(33.3)	2(7.7)	0.048*	9(56.2)	4(17.4)	0.011*	8(57.1)	3(50.0)	1.000
Live birth /ET (%)	8(28.6)	15(41.7)	0.279	11(28.9)	23(41.8)	0.205	7(12.3)	19(25.7)	0.057	6(6.8)	3(4.7)	0.735
Number of surplus vitrified embryo	37	16		85	19		65	17		61	3	
Surplus vitrified embryo /patient Mean (SD)	1.32 (1.83)	0.44 (0.73)	0.011*	2.24 (2.60)	0.35 (0.73)	< 0.001*	1.14 (1.44)	0.23 (0.75)	< 0.001*	0.69 (1.36)	0.05 (0.28)	0.001*
Cumulative live birth /ITT (%)	13(46.4)	16(44.4)	0.874	15(39.5)	24(43.6)	0.689	8(14.0)	20(27.0)	0.072	7(8.0)	3(4.7)	0.520
Cumulative live birth /OPU (%)	13(11.1)	16(44.4)	< 0.001*	15(7.8)	24(43.6)	< 0.001*	8(2.7)	20(27.0)	< 0.001*	7(1.3)	3(4.7)	0.086

Data are mean ± standard deviation or n (%) and compared among groups using Student’s *t-*test, chi-square test, or Fisher’s test for *P*-value. Abbreviations: *Accu* accumulation of vitrified oocytes; fresh = Fresh oocytes; *DOR* diminished ovarian reserve, *OPU* ovum pick-up, *ART* assisted reproduction technology, *M-II* metaphase II, *ET* embryo transfer, *ITT* intention to treat

^*^ *p* < 0.05

Table 3 shows the clinical outcome of patient-accumulated vitrified M-II oocytes reaching the goal of a total number ≥ 10 and ≥ 15. More mean number of available M-II oocytes to create more embryos for ET in the DOR-Accu group contribute to increasing CPR (48.4% vs. 31.0%, *p* = 0.054), but it fail to improve LBR per ET (29.0% vs. 26.2%, *p* = 0.738) and CLBR per ITT (29.0% vs. 27.5%, *p* = 0.859). Therefore, higher MR (40.0% vs. 14.1%, *p* = 0.03) in the DOR-Accu group was still notable.

**Table 3:** Clinical outcomes of the strategy for managing DOR compared between fresh oocytes and accumulated vitrified oocytes reach ≥ 10 or ≥ 15 M-II oocytes

	DOR-fresh (A)	DOR-Accu		*p*	
		≥ 10 M-II (B)	≥ 15 M-II (C)	A vs B	A vs C
Number of patients	229	100	31		
Number of OPU	229	654	256		
OPU /patient Mean (SD)	1.00(0.00)	6.54(3.04)	8.26(3.60)	< 0.001*	< 0.001*
Maternal age at ART start Mean (SD)	38.07(3.77)	39.75(3.78)	39.26(3.61)	< 0.001*	0.098
AMH Mean (SD)	0.72(0.32)	0.57(0.34)	0.67(0.32)	< 0.001*	0.376
M-II oocytes /patient Mean (SD)	3.53(1.57)	13.57(4.14)	18.61(3.79)	< 0.001*	< 0.001*
Fertilization of fresh and survival egg % (SD)	75.29(24.69)	71.88(18.37)	73.31(12.91)	0.215	0.662
Number of the transfer cycle	229	100	31		
Number of embryos transferred Mean (SD)	2.14(0.87)	3.26(0.82)	3.26(0.86)	< 0.001*	< 0.001*
Pregnancy /ET (%)	71(31.0)	36(36.0)	15(48.4)	0.374	0.054
Implantation rate % (SD)	17.47(29.52)	18.08(28.58)	26.34(34.57)	0.861	0.125
Miscarriage /pregnancy (%)	10(14.1)	15(41.7)	6(40.0)	0.001*	0.030*
Stillbirth /pregnancy (%)	1(1.7)	0(0.0)	0(0.0)	1.00	1.00
Live birth /ET (%)	60(26.2)	21(21.0)	9(29.0)	0.314	0.738
Number of surplus vitrified embryo	55	179	85		
Surplus vitrified embryo /patient Mean (SD)	0.24(0.65)	1.79(2.18)	2.74(2.58)	< 0.001*	< 0.001*
Cumulative live birth /ITT (%)	63(27.5)	26(26.0)	9(29.0)	0.777	0.859
Cumulative live birth /OPU (%)	63(27.5)	26(4.0)	9(3.5)	< 0.001*	< 0.001*

Data are mean ± standard deviation or n (%) and compared among groups using Student’s *t-*test, chi-square test, or Fisher’s test for *P*-value. Abbreviations: *DOR-fresh* diminished ovarian reserve, fresh oocytes, *DOR-Accu* diminished ovarian reserve, accumulation of vitrified oocytes, *DOR* diminished ovarian reserve, *M-II* metaphase II, *OPU* ovum pick-up, *ART* assisted reproduction technology, *AMH* Anti-Müllerian hormone, *ET* embryo transfer, *ITT* intention treat

^*^ *p* < 0.05

Table 4 evaluated whether age, AMH, managing DOR strategy, and the number of embryos transferred affect clinical outcomes. Although the maternal age of ART start and ET were older and AMH was lower in the DOR-Accu group compared with the DOR-fresh group (Table 1), it did not affect clinical outcomes. However, a vitrified oocyte accumulation strategy negatively affected effects on MR per pregnancy (OR: 4.00, 95% CI = 1.10–14.58) and LBR per ET (OR: 0.42, 95% CI = 0.20–0.89). LBR per ET improved as more embryos transfer: 2 embryos (OR: 3.40, 95% CI = 1.41–8.18), 3 embryos (OR: 3.19, 95% CI = 1.25–8.10), and 4 embryos (OR: 5.94, 95% CI = 1.99–17.71).

**Table 4:** The effect of the relevant significant variables on clinical outcomes

	CPR Adj-OR (95%CI)	*p*	MR Adj-OR (95%CI)	*p*	LBR Adj-OR (95%CI)	*p*
Maternal age at ET^a^	0.71 (0.46–1.10)	0.125	0.94 (0.42- 2.12)	0.884	0.84 (0.50–1.42)	0.523
Maternal age at ART start^a^	1.18 (0.77–1.82)	0.448	1.44 (0.65–3.15)	0.368	0.97 (0.58–1.64)	0.914
AMH^a^	0.81 (0.41–1.63)	0.561	0.78 (0.17–3.47)	0.739	0.98 (0.44–2.17)	0.955
Strategy^b^		0.478		0.036*		0.022*
Fresh IVF	1		1		1	
Accumulated vitrified M-II	0.80 (0.44–1.48)	0.478	4.00 (1.10–14.58)	0.036*	0.42 (0.20–0.89)	0.022*
Number of Embryos transferred^b^		< 0.001*		0.327		0.013*
1	1		1		1	
2	4.23 (1.80–9.97)	0.001*	0.00 (0.00-Inf)	0.99	3.40 (1.41–8.18)	0.006*
3	6.48 (2.67–15.72)	< 0.001*	0.00 (0.00-Inf)	0.99	3.19 (1.25–8.10)	0.015*
4	9.07 (3.30–24.91)	< 0.001*	0.00 (0.00-Inf)	0.99	5.94 (1.99–17.71)	0.001*

Linear regression was used to analyze continuous variables and logistic regression was used to evaluate categorical variables. Each variable was adjusted for the strategy for managing DOR, AMH, the number of embryos transferred, ET day, maternal age at ET, and ART start. Abbreviations: *CPR* clinical pregnancy rate, *MR* miscarriage rate, *LBR* live birth rate, *ET* embryo transfer, *ART* assisted reproduction technology, *AMH* Anti-Müllerian hormone, *IVF* in vitro fertilization, *M-II* metaphase II, *ET* embryo transfer

^*^p < 0.05

^a^Continuous variables

^b^Categorical variables

## Discussion

This study showed that DOR women who used the vitrified oocyte accumulation strategy to obtain more embryos for transfer failed to improve CPR. Statistically, higher MR results in lower LBR per ET. More surplus vitrified embryos did not also improve CLBR.

Vitrification-thawing oocytes presented an 85.7% survival rate. Based on the survival rates of thawing oocytes described in previous studies [4, 6–14, 16, 18, 20, 21, 34], we concluded that the vitrification-thawing program had been standardized. Vitrified oocytes are a mature technology for reproduction preservation and have similar outcomes to fresh oocytes in donor women [6–16]. But intracellular ice crystals formation, solution effects, and osmotic shock, which cause oocytes damage, still exit during cryopreservation. DNA fragmentation, chromosome disorganization, aberrant gene expression, and damage to mitochondria, endoplasmic reticulum, and lysosomes have also been found in oocytes after cryopreservation [25–27, 35–40]. The meiotic spindle is a determinant of oocyte viability. Poor spindle architecture impact chromosome stability, fertilization, and possible embryonic development and results in high aneuploidy levels, which cause embryo degeneration and spontaneous abortion [41–47]. Disappearance and reappearance of the meiotic spindle occur during the cooling-thawing procedure, and temperature fluctuations as small as 0.3 °C for short times can cause irreversible spindle damage [47–50]. Aberrant spindles are frequently found in oocytes obtained from women of advanced reproductive age [44, 45], and it may cause oocytes from older women who are more vulnerable to cryopreservation damage. Our results showed that women over 35 in the DOR-Accu group have a relatively high abortion rate compared with the DOR-fresh group. It may approve this speculation. Spindle architecture in oocytes from women younger than 35 is healthy and suffers less damage from cooling and thawing. Therefore, MR was comparable to fresh oocytes.

We set the goal of accumulating the total number of 10–15 M-II oocytes in the DOR-Accu group, which was expected to get higher CPR than in the fresh oocyte cycle. However, only 100 patients (47.4%) accumulated vitrified oocytes reached a total number of ≥ 10, and 31 patients (14.7%) achieved a total number of ≥ 15. It can be attributed to DOR that yielded low oocytes count despite double stimulation in the same ovarian cycle, maximizing oocyte output [29, 30]. Patients need to receive repeat ovarian stimulation and retrieval 8.26 times to reach a total number ≥ 15 of accumulation. Although the vitrified oocyte accumulation strategy may palliate DOR women the psychological distress caused by repeated transfer failures [4], they are still distressed from stimulation cancellation, repeat invasive procedures, and failure retrieving, which discourage them from accumulating enough vitrified M-II oocytes. The cost and risk of repeat ovarian stimulation and *retrieval could* be higher in the DOR-Accu group, even in mild COS with flexible gonadotropin use. Even DOR women who accumulate vitrified M-II oocytes reach ≥ 15 and create more embryos to improve CPR. Higher MR (40%) counterbalances it and results in similar LBR and CLBR per ITT compared with the DOR-fresh group. Although vitrified M-II oocytes need an average of 6–9 times of OPU to get similar LBR and CLBR to 1 IVF cycle using fresh oocytes, it is a poorly cost-effective strategy for managing DOR. Previous studies showed that the vitrified oocyte accumulation strategy inseminated vitrified oocytes that pooled together with fresh oocytes got similar outcomes compared with the IVF cycle using fresh oocytes [4, 5]. More poor outcomes per ET or OPU are expected if only accumulated vitrified oocytes were used.

It is noteworthy that CLBR was poor in both groups as women aged over 40 (8.0% vs. 4.7%, *p* = 0.52). It is not surprising because previous research reported that CLBR worsened dramatically after the age of 40 years [51, 52], and our data showed similar results. CLBR was poor in both groups after 37 years, regardless of the number of accumulating vitrified M-II oocytes. Moreover, 28 patients who underwent 229 ovum retrievals and harvested 170 oocytes, and these extremely DOR women who obtained less than the mean one of M-II oocytes per OPU, all got no live birth finally*.* However, we need to extend the sample size and perform a randomized controlled trial to approve observation results and make the conclusion.

Our study has some limitations. It is a retrospective review of patients who had obtained oocytes from retrieval in both groups, and we did not include patients who had no embryos transferred for any cause. We did not calculate the cycle cancellation and patient dropout rates and just focused on transfer outcomes.

It pointed out that the average age at ART start and ET was older, and average AMH serum levels were lower in the DOR-Accu group. However, these differences between groups were too minimal to confound clinical outcomes, which was also approved in Table 4. Moreover, statistically higher MR (40.0% vs. 14.1%, *p* = 0.03), similar LBR (29.0% vs. 26.2%, *p* = 0.738), and CLBR (29.0% vs. 27.5%, *p* = 0.738) exist in subgroup of patient accumulated vitrified oocytes reach ≥ 15 M-II oocytes despite similar age at ART start and AMH serum levels in two groups.

## Conclusion

Our result demonstrated that accumulation of oocytes by vitrification for DOR women fails to improve LBR even if accumulation reaches a total of ≥ 15 vitrified M-II oocytes. Moreover, higher MR (41.4%) in the DOR-Accu group resulted in lower LBR (15.2% vs. 26.2%, *p* = 0.004). It is difficult to accumulate vitrified M-II oocytes to reach a total of ≥ 15 because only 14.7% achieved the goal. Even if patients reach this goal of accumulating vitrified M-II oocytes, it took an average of 8.26 times OPU to get similar LBR per ET and CLBR from 1 IVF cycle using fresh oocytes. Accumulating vitrified M-II oocytes is less efficient and has lower efficacy than IVF cycles using fresh oocytes for managing DOR.

## Data Availability

The datasets used and/or analyzed during the current study are available from the corresponding author on reasonable request.

## Abbreviations

LBR: Live birth rate
DOR: Diminished ovarian reserve
AMH: Anti-Müllerian hormone
AFC: Antral follicle count
DOR-Accu: Diminished ovarian reserve accumulation of vitrified oocytes
ET: Embryo transfer
COS: Controlled ovarian stimulation
DOR-fresh: Diminished ovarian reserve fresh oocytes
CLBR: Cumulative LBR
ITT: Intention to treat
CPR: Clinical pregnancy rate
MR: Miscarriage rate
M-II: Metaphase II
POR: Poor ovarian responders
IVF: In vitro fertilization
ART: Assisted reproduction technology
GnRH: Gonadotropin-releasing hormone
VS: Vitrification solution
TS: Warming solution
WS: Washing solution
ICSI: Intracytoplasmic sperm injection
E2: Estradiol
IR: Implantation rate
OR: Odds ratios
Cis: Confidence intervals
